# Harnessing Bacterial Signals for Suppression of Biofilm Formation in the Nosocomial Fungal Pathogen *Aspergillus fumigatus*

**DOI:** 10.3389/fmicb.2016.02074

**Published:** 2016-12-22

**Authors:** F. Jerry Reen, John P. Phelan, David F. Woods, Rachel Shanahan, Rafael Cano, Sarah Clarke, Gerard P. McGlacken, Fergal O’Gara

**Affiliations:** ^1^BIOMERIT Research Centre, School of Microbiology, University College Cork – National University of IrelandCork, Ireland; ^2^Department of Chemistry and Analytical and Biological Chemistry Research Facility, University College Cork – National University of IrelandCork, Ireland; ^3^School of Biomedical Sciences, Curtin Health Innovation Research Institute, Curtin University, PerthWA, Australia

**Keywords:** biofilm, *Aspergillus fumigatus*, *Pseudomonas aeruginosa*, interkingdom interactions, alkylhydroxyquinolone (AHQ), PQS, HHQ

## Abstract

Faced with the continued emergence of antibiotic resistance to all known classes of antibiotics, a paradigm shift in approaches toward antifungal therapeutics is required. Well characterized in a broad spectrum of bacterial and fungal pathogens, biofilms are a key factor in limiting the effectiveness of conventional antibiotics. Therefore, therapeutics such as small molecules that prevent or disrupt biofilm formation would render pathogens susceptible to clearance by existing drugs. This is the first report describing the effect of the *Pseudomonas aeruginosa* alkylhydroxyquinolone interkingdom signal molecules 2-heptyl-3-hydroxy-4-quinolone and 2-heptyl-4-quinolone on biofilm formation in the important fungal pathogen *Aspergillus fumigatus*. Decoration of the anthranilate ring on the quinolone framework resulted in significant changes in the capacity of these chemical messages to suppress biofilm formation. Addition of methoxy or methyl groups at the C5–C7 positions led to retention of anti-biofilm activity, in some cases dependent on the alkyl chain length at position C2. In contrast, halogenation at either the C3 or C6 positions led to loss of activity, with one notable exception. Microscopic staining provided key insights into the structural impact of the parent and modified molecules, identifying lead compounds for further development.

## Introduction

*Aspergillus fumigatus* is an opportunistic pathogenic fungus associated with hospital-acquired infections. *A. fumigatus* infections contribute to morbidity and mortality in people with respiratory diseases such as cystic fibrosis (CF) and chronic obstructive pulmonary disease (COPD), as well as infecting open skin wounds in burn patients and medical implants (Peleg et al., 2010; Speirs et al., 2012). Indeed, there is a growing awareness of the importance of fungal pathogens in the lung and other sites of infection through global microbiome analyses (Kolwijck and van de Veerdonk, 2014; Kim et al., 2015). However, as with their bacterial counterparts, function and effect in relation to their contribution to the pathophysiology of disease can be harder to define. *Aspergillus* infection takes two principal forms; invasive pulmonary aspergillosis (IPA) and chronic necrotising pulmonary aspergillosis, with the latter being characterized by the transformation of conidia to hyphae (germination), which can subsequently invade the tissue (Kaur and Singh, 2014; Kolwijck and van de Veerdonk, 2014). Hyphal stage cells typically adopt a structured biofilm, either as monospecies or multispecies, at which point the biofilm-embedded cells become highly resistant to antimicrobial drug therapy (Seidler et al., 2008; Manavathu et al., 2014). Several reasons have been proposed to explain this increased tolerance, such as biofilm specific upregulation of eﬄux proteins, the presence of extracellular matrices, and persister cells that are inherently drug resistant/tolerant due to their low metabolic rate (Manavathu et al., 2014).

These inherently resistant biofilm characteristics of *A. fumigatus* have led to conventional antifungal therapies rapidly becoming redundant in the treatment of not only nosocomial infections (e.g., hematologic disease) but also on indwelling medical devices (IMDs) and implants (Ramage and Williams, 2013). While both *Candida* and *Aspergillus* species are causative agents in approximately 8% of all implant infections, their potency and resistance to antifungals is increasing steadily with survival rates for some implant patient groups as low as 50% (Lynch and Robertson, 2008; Ramage and Williams, 2013). More recently, *Aspergillus* contamination of nebuliser devices used by CF patients has been reported (Peckham et al., 2016). The upward trend in Aspergillosis and other major fungal infections poses a significant clinical, financial and health threat and requires concerted and sustained efforts to identify and characterize novel anti-fungal agents to tackle these preventable diseases.

It is now becoming clear that the co-existence and mutual interaction of bacteria and fungi at the site of infection contributes to the pathogenesis of disease. In the case of CF, *A. fumigatus* has been isolated in up to 60% of patients with *Pseudomonas aeruginosa* infection, suggesting a close relationship between established colonization by *P. aeruginosa* and superinfection by *A. fumigatus* (Paugam et al., 2010; Briard et al., 2015). Interkingdom interactions between *A. fumigatus* and bacterial competitors has been described previously, with significant attention focused on the interaction with *P. aeruginosa* (McAlester et al., 2008; Mowat et al., 2010; Briard et al., 2015; Ferreira et al., 2015; Zheng et al., 2015). Perhaps somewhat surprisingly, given their co-occurrence, many of these studies describe an antagonistic influence of *P. aeruginosa* secreted factors on *A. fumigatus* biofilm formation. Mowat et al. (2010) ascribed the competitive inhibition of filamentous *A. fumigatus* growth by *P. aeruginosa* to the effects of small diffusible and heat-stable molecules. Interestingly, anti-biofilm activity in the supernatants of *P. aeruginosa lasR* and *lasI* quorum sensing mutants was reduced but not lost entirely, suggesting the factors responsible are not entirely under Acyl Homoserine Lactone (AHL) control. Several studies have described the impact of redox active metabolites produced by *P. aeruginosa* on biofilm formation and hyphal development in *A. fumigatus* (Briard et al., 2015; Zheng et al., 2015), effects that are likely to be strain and condition specific (Ferreira et al., 2015). More recently, Briard et al. (2016) demonstrated stimulation of *A. fumigatus* growth by volatile compounds produced by *P. aeruginosa*. Moreover, strain specificity was also highlighted in a recent study by Shirazi et al. (2016) where biofilm filtrates of *P. aeruginosa* strains isolated from CF patients were shown to inhibit pre-formed *A. fumigatus* biofilms through apoptosis. Therefore, it is clear that the factors that govern the dynamics of the *P. aeruginosa – A. fumigatus* interaction *in vivo* remain to be fully understood. From a clinical perspective, a strategy that targets biofilm inhibition should be less susceptible than conventional antibiotic treatment to the emergence of resistance in pathogens, particularly as metabolic processes and bacterial growth are not directly targeted.

In this study, we investigated the anti-biofilm activity of *P. aeruginosa* alkylhydroxyquinolone (AHQ) autoinducers against *A. fumigatus*. Growth was unaffected, while biofilm biomass and structure was significantly altered in the presence of both 2-heptyl-4-quinolone (HHQ) and 2-heptyl-3-hydroxy-4-quinolone (PQS). Having established the activity of the parent molecules, a suite of analogs was tested for anti-biofilm activity and lead compounds were identified that retained activity against *A. fumigatus*. The synthesis of new compounds incorporating decorations on the anthranilate ring identified further lead compounds with activity comparable to the parent HHQ molecule, providing important insights into the structure function relationship underpinning the suppression of fungal biofilms. Importantly, anti-biofilm activity was retained against clinical isolates from a pediatric cohort, providing a framework for further functionalization and future clinical implementation.

## Materials and Methods

### Fungal and Bacterial Culture Conditions

*Aspergillus fumigatus* Af293 was routinely grown on Sabouraud dextrose agar (SDA) in 100 ml cell culture flasks at 37°C for 3–4 days. Sputum samples from a pediatric CF cohort (Cork University Hospital) were cultured on SDA plates and incubated for 4–7 days at 37°C. Conidia were collected by scraping in PBS with 0.025% (v/v) Tween 20, and the Internal Transcribed Spacer (ITS) region was amplified by PCR using ITS4 (5′-TCCTCCGCTTATTGATATGC-3′) and ITS5 (5′-GGAAGTAAAAGTCGTAACAAGG-3′) for taxonomic identification. PCR reaction conditions were as follows: 30 cycles of alternating denaturation (94°C for 45 s), annealing (58°C for 45 s), and extension (72°C for 2 min). Yeast cultures of *Candida albicans* were routinely grown on yeast peptone dextrose (YPD) agar and were cultured at 30°C overnight. Bacterial cultures of *P. aeruginosa* containing the pLP0996 *pqsA-lacZ* promoter fusion were maintained on Luria Bertani (LB) plates supplemented with carbenicillin (200 μg/ml), and routinely grown at 37°C.

### Spore Capture and Biofilm Assay

Conidia were harvested by flooding the surface of the agar plates with 5 ml PBS (Oxoid) containing 0.025% (v/v) Tween 20 and gently moving the liquid over the surface of the fungal lawn. The conidial suspension was transferred into a 25 ml sterile container and conidia were counted using a Neubauer haemocytometer and light microscope. Conidia were adjusted to the required concentration in RPMI 1640 (Sigma) buffered to pH 7.0 with 0.165 M MOPS immediately prior to biofilm formation analysis.

To assess biofilm formation, counted *A. fumigatus* spores (1 × 10^5^) in MOPS buffered RPMI 1640 were inoculated into 24- and 96-well plates and grown overnight in the presence of 100 μM HHQ, PQS and the compound collection. After 24 h, the media was removed and the biofilm washed twice with distilled water after which 0.1 % crystal violet (CV) was added to each well and allowed to stand for 1 h at room temperature. The CV was removed and all wells washed in a water bath by inversion. Ethanol was added to each well to solubilize biofilms after which samples were read on a spectrophotometer at OD_595nm_.

Analysis of preformed biofilms (24 h) was carried out by allowing biofilms to establish in buffered RPMI, or compound/carrier treated RPMI as described above. Disks were removed and washed gently in PBS to remove unattached cells. Disks from the buffered RPMI wells were transferred into 1 ml of buffered RMPI with 100 μM HHQ, **1**, or methanol control. In tandem, disks from pre-treated wells were also transferred and treated again with their respective compound or methanol control. All plates were incubated for 24 h at 37°C and biofilm quantified as before.

### Staining and Microscopy

*Aspergillus fumigatus* spores (1 × 10^5^) were inoculated onto plastic coverslips in 24 well plates and grown in the presence of analogs overnight at 37°C in RPMI buffered with MOPS pH 7.0. After 24 h, plastic coverslips were washed once in PBS and stained. Calcofluor (1 mg/ml) and 10% (w/v) potassium hydroxide were added drop-wise to coverslips, washed in PBS, mounted, and viewed. Concanavalin A (Con A) and FUN-1 were added at 50 μg/ml in 1 ml PBS and incubated at 37°C for 30 min. All imaging was carried out on a Zeiss LSM5 confocal microscope. Confocal images were recorded under a bright field lens using ×20 objective magnification. Filter cubes facilitating fluorescent imaging were used to record images for Calcofluor at Abs_405nm_, Con A at Abs_488nm_ and FUN-1 at Abs_543nm_. All images were captured using the Zeiss HBO-100 microscope illuminating system, processed using the Zen AIM application imaging program and converted to JPG using Axiovision 40 Ver. 4.6.3.0.

### Growth Assays

*Aspergillus fumigatus* Af293 was grown in RPMI buffered media or YPD to assess sensitivity to HHQ, PQS, and lead analogs. Spore suspensions were collected from SDA plates in PBS Tween 20 (0.025%), counted using a haemocytometer, and inoculated into fresh YPD to a cell count of 1 × 10^5^ spores/ml. Spore suspensions, either treated with lead compounds, carrier solvent, or untreated, were then added in quadruplicate to 96-well plates and incubated with gentle agitation for 24 h. The OD_405nm_ was determined spectrophotometrically.

### Promoter Fusion Analysis

Alkylhydroxyquinolone signaling was monitored using the *pqsA-lacZ* promoter fusion pLP0996 in both the wild-type PAO1 strain and its isogenic *pqsA^-^* mutant (McGrath et al., 2004). Cells from overnight cultures were transferred to fresh LB media (carbenicillin 200 μg/ml) to a starting OD_600nm_ 0.02 in the presence of compounds 20–24 (100 μM) or equivalent volumes of DMSO. Samples were taken at late log phase growth and β-galactosidase activity was measured as before (Reen et al., 2016).

### Cytotoxicity Assays

Lactate dehydrogenase (LDH) release from HeLa (cervical epithelial adenocarcinoma) cells was assayed as a measure of cytotoxicity using an LDH colorimetric kit (Roche) according to manufacturers’ instructions. HeLa cells were seeded into 96 well plates and treated with methanol (control) and analogs. Following a 16 h incubation at 37°C and 5% CO_2_, supernatants were removed and added to catalyst reaction mixture in a fresh plate and further incubated at 37°C and 5% CO_2_ for 30 min to allow for color development. After this period, the plate was analyzed by measuring the absorbance on a microplate spectrophotometer at OD_490nm_.

### XTT Biofilm Assays

The *C. albicans* biofilm response to analogs was assessed using the XTT [2,3-Bis-(2-Methoxy-4-Nitro-5-Sulfophenyl)-2H-Tetrazolium-5-Carboxanilide] assay in 96 well plates at 37°C. After overnight growth in the presence of methanol (control) and analogs, *C. albicans* biofilms were treated with 100 μl freshly prepared XTT (Sigma) and menadione (Sigma). After a 2 h incubation at 37°C, biofilm formation was determined by measuring the absorbance at OD_492nm_ on a microplate spectrophotometer.

### Statistical Analysis

Data presentation and statistical analysis was performed using the GraphPad Prism v5.03 software package or the Bootstratio algorithm (Cleries et al., 2012).

### Compound Synthesis

The synthesis of HHQ, PQS (Pesci et al., 1999; McGlacken et al., 2010) and other HHQ-based analogs (Reen et al., 2012, 2015, 2016) was carried out via previously described methods. Novel compounds and compounds which required modified syntheses are described *vide infra* and in the Supplementary Data Sheet.

### Ethics Statement

Sputum samples were collected from pediatric patients attending the CF clinic at Cork University Hospital, Ireland. This study was carried out in accordance with the recommendations of the Clinical Research Ethics Committee of the Cork Teaching Hospitals (CREC), with written informed consent from all subjects. All subjects gave written informed consent in accordance with the Declaration of Helsinki. The protocol was approved by the CREC and all samples were handled in accordance with the approved guidelines.

## Results

### *P. aeruginosa* Signal Molecules Interfere with Biofilm Formation in *A. fumigatus*

**FIGURE 1:**
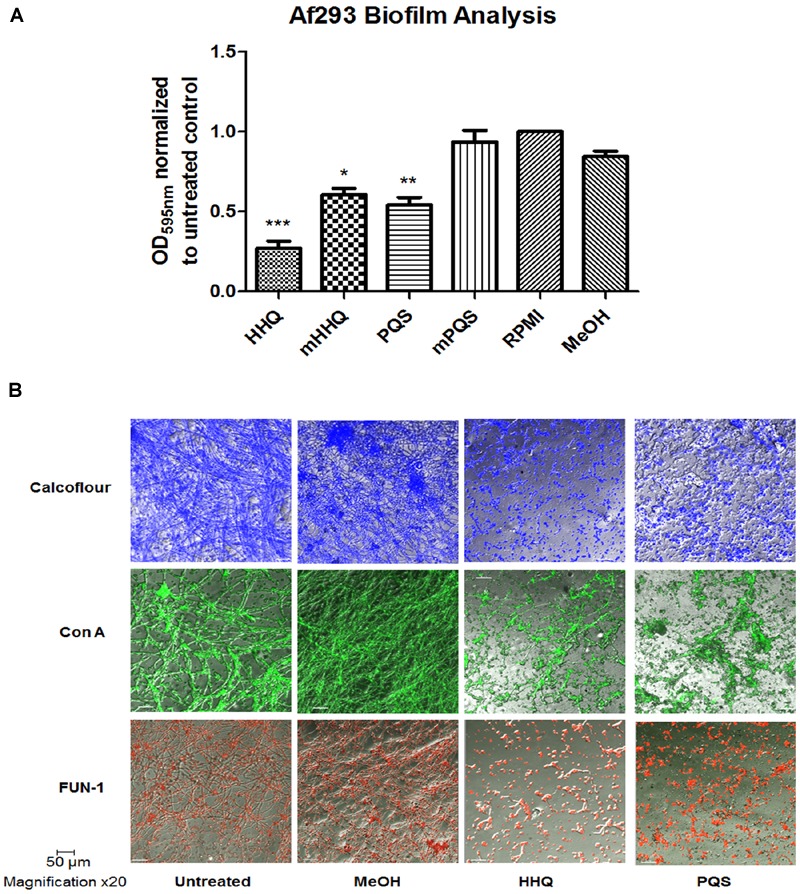
**Suppression of *A. fumigatus* biofilm formation in response to HHQ and PQS. (A)** Biofilm assays using crystal violet (CV) staining. mPQS and mHHQ refer to the parent compounds with the C7 alkyl chain removed. RPMI constitutes the untreated sample whereby no compound or carrier has been added. Data is presented as mean fold change (+/- SEM) of Abs_595nm_ relative to untreated cells. Data is representative of five independent biological replicates. Statistical significance using methanol treated cells as reference is represented by one way ANOVA with *post hoc* Bonferroni correction (^∗^*p* < 0.05, ^∗∗^*p* < 0.005; ^∗∗∗^*P* < 0.001). **(B)** Confocal microscopic analysis (×20) of biofilm formation on plastic coverslips. Staining was performed using Calcofluor-1 (cellulose and chitin), Concanavalin A (live cellular vacuoles), and FUN-1 (lectins/carbohydrates).

The *P. aeruginosa* signaling molecules HHQ and PQS have previously been shown to possess interkingdom activity, influencing the phenotypic and transcriptional behavior of both microbes and higher order organisms (Reen et al., 2011; Tashiro et al., 2013; Trejo-Hernandez et al., 2014), including mammalian cells (Kim et al., 2010). While the *P. aeruginosa* secretome has previously been shown to suppress biofilm formation in *A. fumigatus* (Mowat et al., 2010), the extent of the active components remained to be defined. Our previous finding that HHQ suppressed biofilm formation in the pathogenic yeast, *C. albicans* (Reen et al., 2011), led us to investigate the anti-biofilm activity of these signals against *A. fumigatus*. Addition of either HHQ or PQS to the model strain *A. fumigatus* Af293 led to a significant reduction in attachment to the wells of polystyrene plates (**Figure 1A**). As expected, loss of the alkyl chain led to a significant reduction in the potency of this anti-biofilm activity, consistent with the functional requirement for this feature in AHQ signaling.

In order to visualize the biofilm structures, attached cells were treated with specific intracellular stains to assess impact on key cellular components. These included cellulose and chitin with Calcofluor; live cellular vacuolar stain FUN-1 which functions as a live/dead stain; and lectin (carbohydrate) structures with Concanavalin A. Confocal microscopic analysis confirmed the effect of HHQ and PQS on formation of biofilm mass and revealed a significant reduction and disruption of hyphal development (**Figure 1B**). Cells treated with either HHQ or PQS appeared locked in a spore form, with minimal evidence for formation of hyphal structures, and the absence of the interwoven network characteristic of *A. fumigatus*.

### Structurally Modified AHQ Analogs Retain Anti-biofilm Activity

**FIGURE 2:**
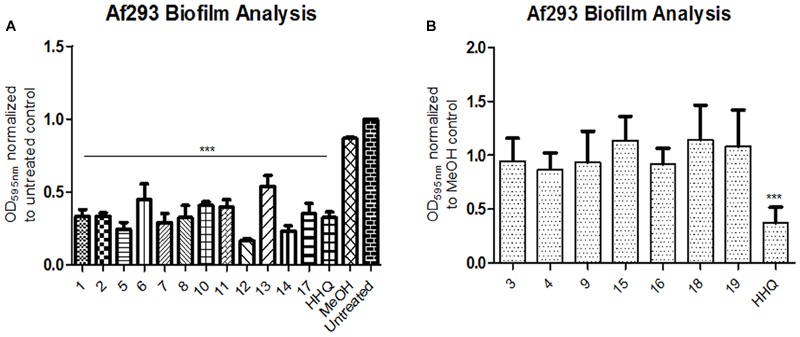
**Biofilm formation in *A. fumigatus* cells following incubation with (A)** active and **(B)** inactive AHQ analogs. CV stained multi-well biofilm assays. Data is presented as mean fold change (+/- SEM) in Abs_595nm_ relative to untreated treated cells and includes at least five independent biological replicates. Statistical significance using methanol treated cells as reference is represented by one way ANOVA with *post hoc* Bonferroni correction (^∗∗∗^*P* < 0.001).

Having established that both HHQ and PQS interfered with biofilm formation, a suite of 4-quinolone analogs, bearing diverse structural features (Supplementary Figure S1), were tested for anti-biofilm activity toward *A. fumigatus*. Analogs included modifications at the C3 and C4 positions, on the anthranilate ring, and of the alkyl chain length, while substitutions were predominantly with halogen, n-hexyl, methyl or methoxy groups. The results of this analysis revealed that an exquisite degree of structural conservation is required for AHQ inter-kingdom activity (**Figure 2A**). Of these, compound **5** (*n*-hexyl at C6) and compound **12** (methoxy at C7) gave the greatest reduction in biofilm formation. Compound **14**, in which the alkyl chain length was extended relative to the parent molecule HHQ, retained its anti-biofilm activity, while addition of halogen groups at C3 led to loss of anti-biofilm activity. Addition of a methoxy (**3**) or methyl (**4**) group to the C6 position of HHQ with a seven carbon alkyl chain led to loss of anti-biofilm activity (**Figure 2B**), while activity was retained in methoxy (**11**) and halogen (**17**) modified equivalents upon extension of the alkyl chain to nine carbons.

**FIGURE 3:**
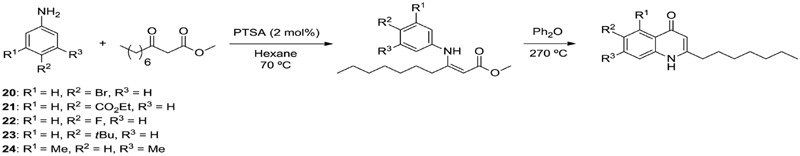
**Synthesis pipeline for anthranilate ring analogs 20–24**.

**FIGURE 4:**
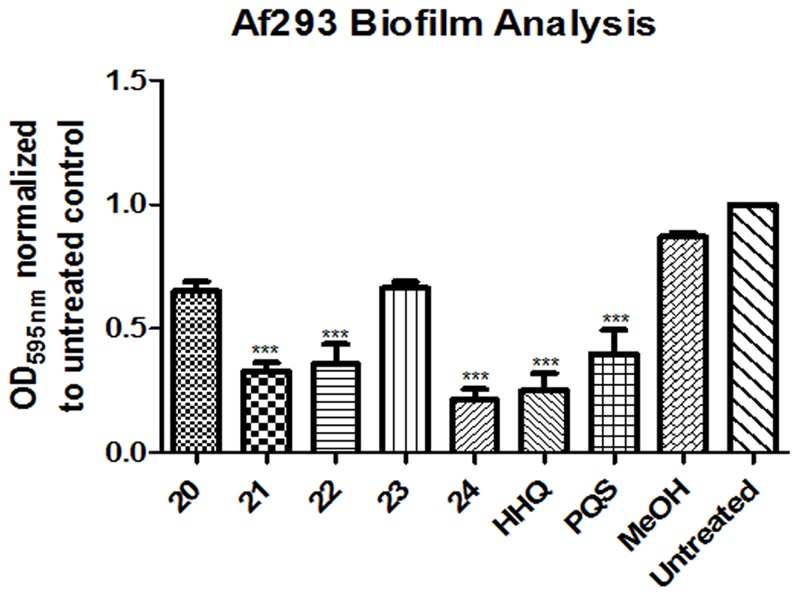
**Anti-biofilm activity of anthranilate ring substituted compounds.** Data is presented as mean fold change (+/- SEM) of Abs_595nm_ relative to untreated cells and includes five independent biological replicates. Statistical significance relative to methanol treated cells is represented by one way ANOVA with *post hoc* Bonferroni correction (^∗^*p* < 0.05; ^∗∗^*p* < 0.005; ^∗∗∗^*P* < 0.001).

Isolation of quinolones with substituents at C5 is difficult, as the corresponding regioisomer (at C7) is also formed in the Conrad-Limpach cyclisation step. Thus we chose to focus on symmetrical aniline precursors, which allowed for further elaboration at the 6-position. Compounds were synthesized with Br (**20**), CO_2_C_2_H_5_ (**21**), an electron withdrawing F (**22**), and an electron donating bulky C–C_3_H_9_ (**23**) at the C6 position (**Figure 3**). Use of the 3,5-dimethyl aniline precursor gave a C5–C7 substituted CH_3_ (**24**). All analogs were assessed for antagonism of biofilm formation in *A. fumigatus* Af293. While two of the substituted analogs, **20** and **23**, had lost their anti-biofilm activity, compounds **21**, **22**, and **24** retained activity against the fungus, suppressing biofilm formation to the same degree as lead compounds (**Figure 4**). It is clear that the structural organization of the anthranilate ring components is of critical importance to the anti-biofilm activity. The retention of anti-biofilm formation by analogs functionalized at the C6 and C8 positions was particularly interesting given that halogenation of these positions had previously been shown to enhance antagonism of PQS signaling in *P. aeruginosa* (Ilangovan et al., 2013).

**FIGURE 5:**
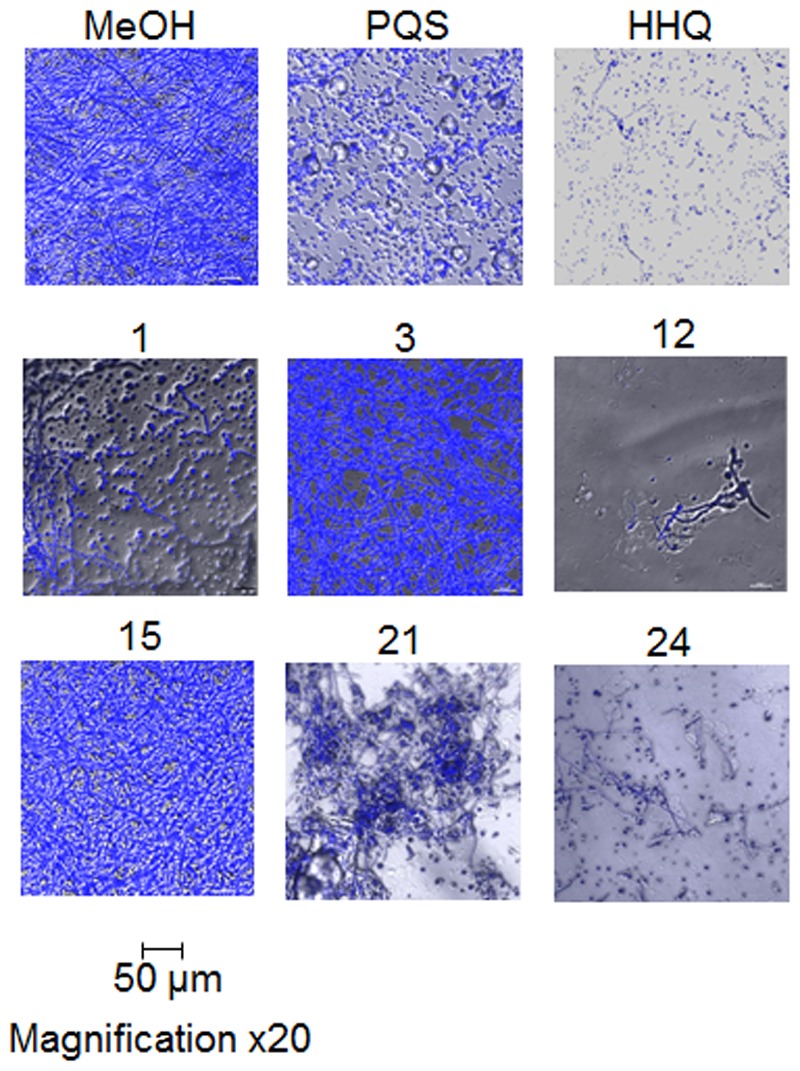
**Microscopic analysis of Af293 biofilm formation on plastic coverslips in the presence of selected active and inactive AHQ analogs**.

Microscopic analysis of biofilms formed on plastic coverslips in the presence of selected compounds confirmed the disruption of hyphal structures in Af293 (**Figure 5**). Staining with Calcofluor revealed the absence of intact hyphal networks in samples treated with compounds **1**, **12**, **21**, and **24** while inactive compounds **3** and **15** did not affect this phenotype. This anti-biofilm activity was shown to be independent of any impact on growth of Af293, with the exception of compound **2** which reduced the final OD_600nm_ reached by 0.1 unit (Supplementary Figure S2).

**FIGURE 6:**
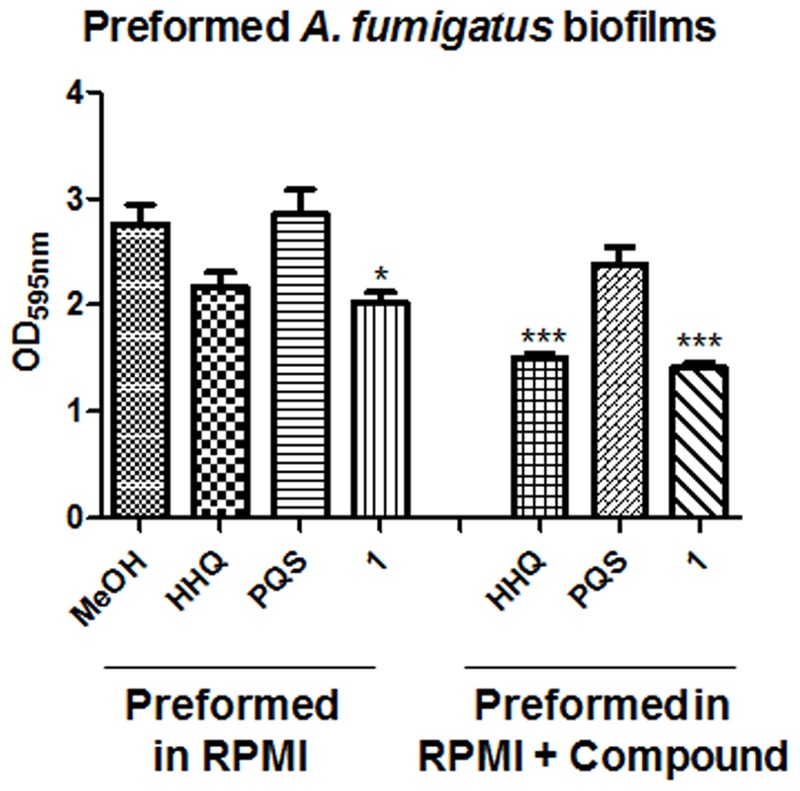
**Anti-biofilm activity of lead compounds on preformed *A. fumigatus* Af293 biofilms on plastic disks.** Data presented is the mean (+/- SEM) of six independent biological replicates. Statistical analysis is performed by one way ANOVA with *post hoc* Bonferronic correction (^∗^*p* < 0.05; ^∗∗∗^*p* < 0.001).

Having determined that the alkylhydroxyquinolone derivative compounds could suppress biofilm formation in *A. fumigatus*, the next stage was to assess whether or not they could affect pre-formed *A. fumigatus* biofilms. The addition of HHQ or **1** to preformed biofilms on plastic disks led to a reduction relative to the methanol control, although only **1** reached statistical significance (**Figure 6**). Pre-treatment from the outset with either HHQ or **1** led to a marked reduction in biofilm formation on disks when compared with the control sample, further evidence that both attachment and the development of hyphal biofilms may be key targets for the lead compounds.

### C6-Modified Analogs Exhibit Non-cytotoxic *A. fumigatus* Specific Activity

**FIGURE 7:**
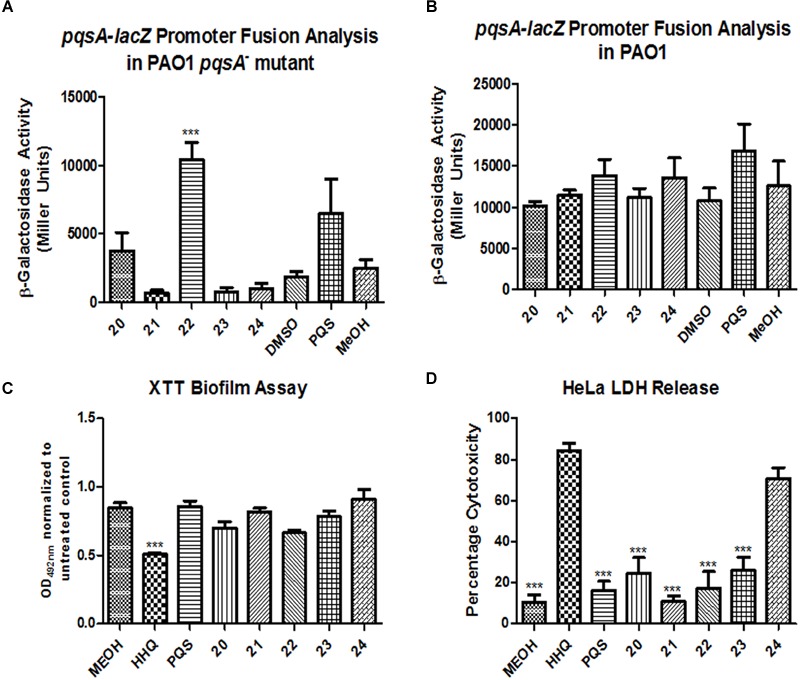
**Profiling the spectrum of activity of compounds 20–24.** Promoter fusion analysis of the *pqsA-lacZ* pLP0996 plasmid in **(A)** a PAO1 *pqsA^-^* mutant, and **(B)** PAO1 wild-type strain, **(C)** XTT biofilm analysis of *C. albicans* and **(D)** LDH release cytotoxicity assays using HeLa cell lines. Data in all experiments is the mean (+/- SEM) of at least three independent biological replicates. Statistical analysis was performed by one-way ANOVA with *post hoc* Bonferroni correction (^∗∗∗^*p* ≤ 0.001).

Any assessment of the utility of these compounds as anti-biofilm agents must consider the origin of the parent molecules HHQ and PQS as co-inducers of AHQ mediated virulence in *P. aeruginosa*. Both HHQ and PQS control multiple virulence phenotypes through activation of the PqsR transcriptional regulator (Xiao et al., 2006). Previously, we have shown that several of the lead analogs described here e.g., **1** and **2** are non-agonists of the AHQ signaling system in *P. aeruginosa*, are selectively non-cytotoxic, and are also effective biofilm blockers in *C. albicans* (Supplementary Table S1) (Reen et al., 2016). Therefore, this analysis was extended to the newly synthesized compounds **20**–**24** to provide a comprehensive profile of all the compounds described in this study. Addition of compounds **21**, **23**, and **24** did not lead to increased *pqsA* promoter activity in a PAO1 *pqsA*^-^ mutant strain indicating that they are non-agonists of the PqsR virulence system (**Figure 7A**). In contrast, compounds **20** and **22** significantly increased expression relative to the control, suggesting that further modification would be required to silence this activity. In spite of being ineffective PqsR ligands, none of the five analogs were capable of interfering with AHQ signaling in the wild-type PAO1 strain, as seen with the comparable activity of the *pqsA* promoter in both treated and untreated cells (**Figure 7B**).

In order to determine whether or not the anti-biofilm activity extends to other fungal pathogens, *C. albicans* was tested for biofilm formation in the presence and absence of compounds **20**–**24** (**Figure 7C**). None of the compounds were found to be active against *C. albicans* biofilm formation, in contrast to some of the other lead compounds identified in this study, such as **1** and **2** (Reen et al., 2016). Further extending the profile and characterization of these compounds, cytotoxicity was measured in a host cell line (**Figure 7D**). Only compound **24** elicited any measurable degree of cytotoxicity, and this was to a lesser extent than the parent molecule HHQ. The other four analogs were comparable to the methanol control.

### AHQ Analogs Block Biofilm in Clinical *A. fumigatus* Isolates

**FIGURE 8:**
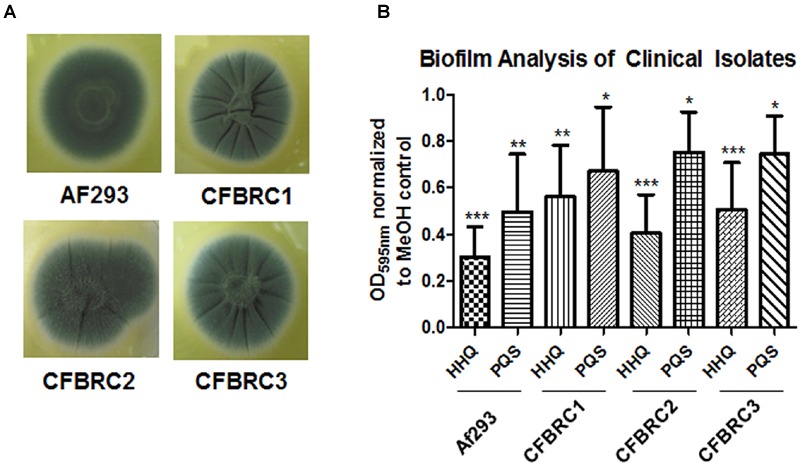
**AHQs suppress biofilm formation in clinical isolates. (A)** Morphological analysis of clinical *A. fumigatus* isolates. **(B)** Biofilm formation in *A. fumigatus* treated cells following incubation with AHQ signals HHQ and PQS. Data is presented as mean fold change (+/- SEM) in OD_595nm_ relative to methanol treated cells and includes five independent biological replicates. Statistical significance is represented by Bootstratio analysis (^∗^*p* < 0.05; ^∗∗^*p* < 0.005; ^∗∗∗^*P* < 0.001).

**FIGURE 9:**
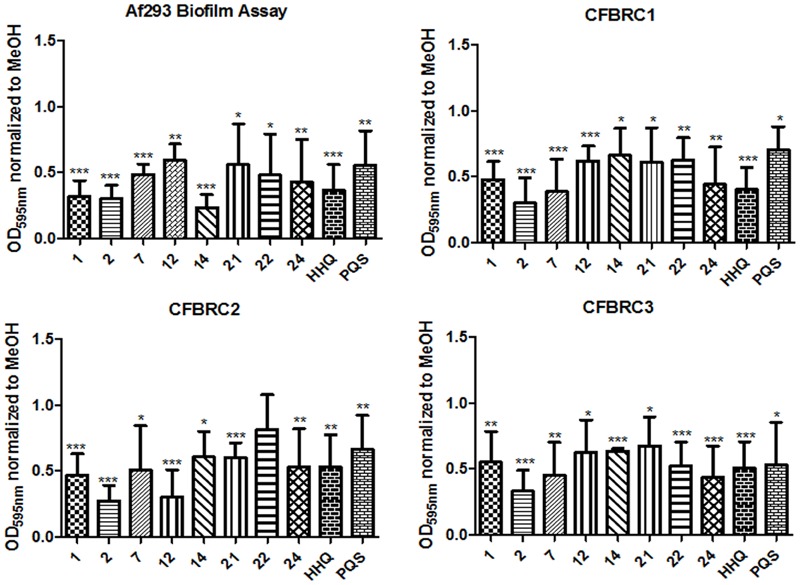
**Biofilm formation in *A. fumigatus* clinical isolates following incubation with AHQ analogs.** Data is presented as mean fold change (+/- SEM) in OD_595nm_ relative to methanol treated cells and includes at least three independent biological replicates. Statistical significance is represented by Bootstratio analysis (^∗^*p* < 0.05; ^∗∗^*p* < 0.005; ^∗∗∗^*P* < 0.001).

There is a growing realization that the genomes of clinical isolates can vary markedly from typed environmental strains, with niche-specific selective pressures manifesting in considerable heterogeneity, even within species. This led us to investigate the effectiveness of the lead compounds against clinical isolates from a pediatric CF cohort, each of which exhibited significant wrinkling of the mycelial mat compared to the smoothness of Af293 (**Figure 8**). Both HHQ and PQS were capable of suppressing biofilm formation in each of the clinical isolates. It was notable, however, that the suppression of biofilm formation was not to same extent as that seen against the lab strain Af293, suggesting some degree of tolerance or adaptation in the clinical isolates (**Figure 8**). Lead compounds were also found to be effective against biofilm formation in clinical strains, underpinning their suitability for further therapeutic development (**Figure 9**).

**FIGURE 10:**
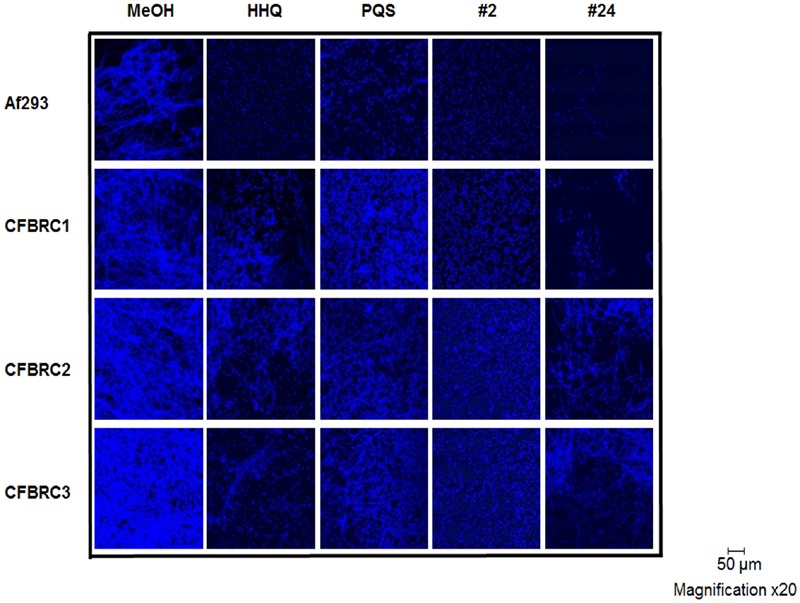
**CLSM analysis of biofilm formation in Af293 and clinical isolates in the presence of lead compounds **2** and **24**.** All data presented is representative of three independent biological replicates.

The structural impact of lead compounds on biofilm formation in both Af293 and the clinical isolates was assessed using confocal microscopy of calcofluor stained cells. As expected, anti-biofilm compounds disrupted the formation of hyphal structures, while inactive compounds (e.g., **23**) did not markedly affect this phenotype. In general, active compounds (e.g., **2** and **24**) appeared to lock the fungal cells in the spore state, with little or no evidence for hyphal development (**Figure 10**). Although present in some of the clinical isolates (e.g., CFBRC2 and CFBRC3, **24**), hyphal structures were weak and sparse, indicating that a key mode of action of these compounds is the prevention of the hyphal switch.

## Discussion

Exposure of immunocompromised individuals to *A. fumigatus* can result in significant morbidity and even mortality in severe cases. The incidence is on the rise owing largely to the increased prevalence of respiratory conditions such as asthma, increased frequency of lung transplantation, and the ongoing use of immunosuppressive therapies. In many of these cases, *A. fumigatus* adopts a biofilm lifestyle, enhancing its persistence and contributing directly to morbidity and mortality associated with these infections. *A. fumigatus* has recently been associated with medical device infections (Ramage et al., 2012; Ramage and Williams, 2013; Nett and Andes, 2015). Cases of serious biomaterial-related infections of joint replacements, catheters, heart valves, cardiac pace makers, and breast augmentation implants have been reported. Often, high risk invasive surgical procedures are used to treat these *A. fumigatus* infections, as the formation of biofilm structures and the associated increased rate of antifungal resistance renders conventional treatment ineffectual (Desai et al., 2014; Nett and Andes, 2015). Antifungal tolerance in fungal infections has been attributed to the secretion of an extracellular polymeric matrix and increased activity of eﬄux pumps (Mowat et al., 2008; Rajendran et al., 2013). Therefore, therapies that combat biofilms directly are likely to enhance the survival and life expectancy of infected individuals.

In this study we have shown that two signal molecules secreted by the nosocomial pathogen *P. aeruginosa* can suppress biofilm formation in *A. fumigatus*. This may be a consequence of the proximity in which these organisms co-exist in clinical and environmental niches, including the lungs of immunocompromised patients and the plant ecosystem. Interactions between co-existing organisms have been widely reported, with signaling molecules showing activity ranging from inter-species to inter-kingdom (Reen et al., 2011; Chen et al., 2014; Michelsen et al., 2014; Kendall and Sperandio, 2016). Both HHQ and PQS were capable of interfering with the formation of *A. fumigatus* biofilms, leading to reduced biomass and the absence of intact hyphal structures in treated cells. Derivatization of the quinolone framework at the C6 and C8 positions revealed the anthranilate ring to be particularly important in the anti-biofilm activity. Several of these compounds have no agonistic activity toward *P. aeruginosa* virulence, unlike the parent molecules HHQ and PQS, thus presenting a viable framework for therapeutic development against medical device fungal pathogens [**Figure 7** and (Reen et al., 2016)]. Furthermore, they are selectively non-cytotoxic to host cell lines, further supporting their development as viable molecular therapeutics. The next stage in the development of these lead compounds will be the characterization of the molecular mechanism(s) through which they elicit their anti-biofilm activity. One approach will be to investigate the possibility of fungal sensory and signal transduction systems that may be involved in perception of alkylhydroxyquinolones and transduction of that response to exist in a non-hyphal growth state. Alternatively, alkyl-quinolones, and PQS in particular, have also been reported previously to possess both pro- and anti-oxidant activity, while interaction with lipopolysaccharide, cellular membranes, and membrane vesicles has also been reported in several bacterial species (Haussler and Becker, 2008; Mashburn-Warren et al., 2009).

**FIGURE 11:**
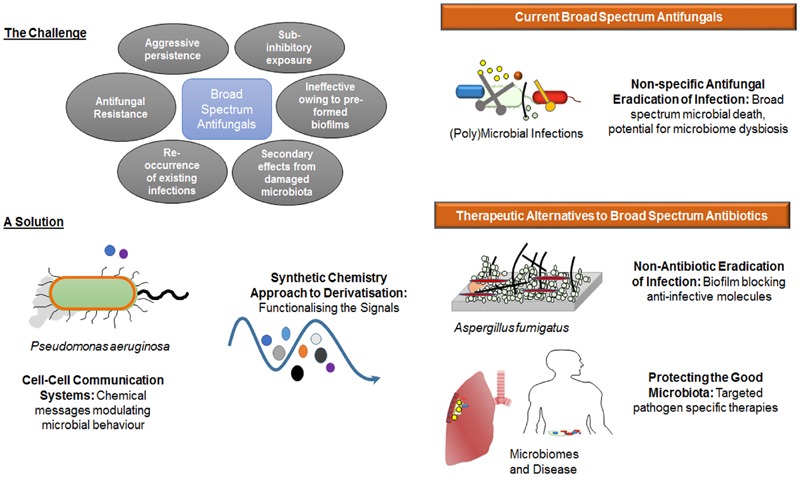
**The lead compounds identified in this study address a key clinical need for alternatives to broad spectrum antifungals.** Challenge: While still the lead option for the management of microbial infections, broad spectrum growth limiting antifungals are increasingly being challenged by limiting factors including antifungal resistance, the aggressive persistence of known pathogens in a biofilm lifestyle, and the collateral damage caused to the host microbiome leading to dysbiosis, Solution: Exploiting new knowledge of cell–cell communication systems and utilizing synthetic chemistry approaches to generate suites of derivatized compounds, has made possible the development of anti-biofilm small molecules that are selectively non-toxic in mammalian cell lines. These have the potential to lock yeast and fungal pathogens such as *A. fumigatus* in the non-hyphal state, potentially rendering them more susceptible to immune challenge, and enhancing the effectiveness of conventional antifungal compounds.

The modulation of *A. fumigatus* biofilm by HHQ and PQS adds another layer of complexity to the interkingdom interactions that are characteristic of mixed *P. aeruginosa* fungal communities. Phenazines, a redox active toxin controlled by PQS and secreted by *P. aeruginosa* reportedly undergo chemical transformation by *A. fumigatus* and *C. albicans* (Moree et al., 2012; Chen et al., 2014). The AHL class of quorum sensing signal has also been shown to play a role in modulating biofilm formation in these fungal pathogens (McAlester et al., 2008; Mowat et al., 2010), while HHQ and PQS have previously been shown to modulate biofilm formation in *C. albicans* (Reen et al., 2011, 2016). Apart from signal mediated interactions, there is recent evidence to suggest that interspecies and interkingdom interactions can foster mutability in co-existing organisms (Michelsen et al., 2014; Trejo-Hernandez et al., 2014). It must also be considered that microbial signals are just one factor in the process of fungal biofilm formation in the host. Other factors such as the availability of nutrients, host related factors such as the immune system and the surface upon which the biofilm will form, mechanical factors such as flow rate within the niche, and the presence of dispersion agents can have a significant influence on fungal biofilm formation. Notwithstanding this, deciphering the chemical messages used by microbes themselves to control the behavior of pathogenic species, without disrupting the growth of the microbiome as a whole, remains a key goal in the age of molecular based therapeutics (**Figure 11**). As a greater understanding of the connection between microbiome dysbiosis and disease status continues to emerge, the critical need for therapeutics that retain the functionality of the core microbiome becomes ever more relevant.

## Conclusion

Microbial communities are founded on intricate and long-evolved communication systems that determine the flux and dynamics of particular species in response to local and environmental cues. Therefore, deciphering these chemical messages has the potential to deliver on the new and innovative alternatives being sought to conventional growth limiting antibiotics. This is an attractive route to therapy due to the chemical tractability of these signals, opening up avenues for functionalization to improve activity and drug-ability of lead compounds. The work described here is highly significant and potentially relevant to several sectors within the clinical and medical device arena where the occurrence of *in vivo* and nosocomial *Aspergillus* infections is destructive from both a human and financial perspective.

## Author Contributions

Conceptualization: FR, GM, and FO. Investigation: FR, JP, DW, RS, RC, and SC. Writing – Original Draft: FR. Writing – Review and Editing: FR, JP, DW, GM, and FG. Funding Acquisition: FR, GM, and FG.

## Conflict of Interest Statement

The authors declare that the research was conducted in the absence of any commercial or financial relationships that could be construed as a potential conflict of interest.
