# Supplementary material for: Harnessing Bacterial Signals for Suppression of Biofilm Formation in the Nosocomial Fungal Pathogen Aspergillus fumigatus
Source: Front Microbiol. 2016 Dec 22;7:2074. doi: 10.3389/fmicb.2016.02074 (PMC5177741; doi:10.3389/fmicb.2016.02074)
Supplement: Supplementary file 1 [file Table_1.DOCX]

| **Compound** | **Agonist Activity*** |
| --- | --- |
| **HHQ** | **+++** |
| **PQS** | **+++** |
| **1** | **-** |
| **2** | **-** |
| **3** | **-** |
| **4** | **+** |
| **5** | **-** |
| **6** | **++** |
| **7** | **-** |
| **8** | **-** |
| **9** | **+++** |
| **10** | **+++** |
| **11** | **-** |
| **12** | **+++** |
| **13** | **+++** |
| **14** | **-** |
| **15** | **+** |
| **16** | **-** |
| **17** | **+** |
| **20** | **+** |
| **21** | **-** |
| **22** | **+++** |
| **23** | **-** |
| **24** | **-** |

**Table S1. Agonist activity of alkylquinolone derivative compounds.**

**Compounds 1 - 17 (Reen *et al* 2016)**

**Compounds 20 - 24 (This study)**

*** Agonist Activity Relative to PQS**

**+++ (80% - 100%)**

**++ (50% - 80%)**

**+ (21% - 50%)**

**- (0 - 21%)**
