# Supplementary material for: Harnessing Bacterial Signals for Suppression of Biofilm Formation in the Nosocomial Fungal Pathogen Aspergillus fumigatus
Source: Front Microbiol. 2016 Dec 22;7:2074. doi: 10.3389/fmicb.2016.02074 (PMC5177741; doi:10.3389/fmicb.2016.02074)

General procedure for the preparation of AHQ analogues

Methyl-3-oxodecanoate

2,2-dimethyl-1,3-dioxane-4,6-dione (Meldrum's acid) (18.7 g, 130 mmol) was dissolved in distilled dichloromethane (200 mL). The solution was cooled to 0°C under a N_2 atmosphere. To the cooled solution were added pyridine (20.5 mL, 260 mmol) and octanoyl chloride (23.8 mL, 140 mmol), drop-wise. The solution was stirred at 0°C for 1 hr and then at room temperature for 1 hr. The mixture was washed with 5% HCl (3 x 75 mL) and with distilled water (75 mL). The solution was then dried with anhydrous $MgSO_4$ filtered and concentrated *in vacuo* to yield acyl Meldrum's acid as a brown oil which was used in the subsequent step without further purification.

Acyl Meldrum's acid was dissolved in MeOH (180 mL) and heated at reflux for 5 hr with constant stirring. After allowing to cool, the reaction mixture was concentrated *in vacuo* yielding the crude product as an orange oil. Purification was achieved by fractional distillation affording the β-keto ester as a pale yellow oil (16.7 g, 64 % yield).

Substituted 2-alkyl-4-quinolones

To a solution of the β-ketoester (5 mmol) in dry hexane (10 mL) were added the substituted aniline (5 mmol) and *p*-toluene sulfonic acid (0.1 mmol). The reaction mixture was heated at reflux (>70°C) under N_2 atmosphere overnight using a Dean-Stark system. Upon completion, the reaction mixture was concentrated *in vacuo* to afford the crude β-enamino ester, which was then added drop-wise to refluxing diphenyl ether (2 mL, >260°C). Reflux was maintained for approx. 1.5 hr. After cooling to room temperature, ether (approx. 20 mL) was added to the reaction mixture and left overnight at 5°C, allowing the quinolone product to precipitate. The quinolone was collected by vacuum filtration, recrystallised from hot methanol (if necessary) and dried *in vacuo*.

Spectra data of new compounds

6-Bromo-2-heptylquinolin-4(1*H*)-one (**20**).
Grey solid; yield: 355 mg (14 %); m. p. = 186-188 ºC (Et_2O); IR (KBr): □□3421, 1632, 1596, 1130 cm^{-1}; ^{1}H-NMR (300 MHz, DMSO-d_6): δ 0.86 (t, *J* = 6.9 Hz, 3H), 1.20-1.35, 1.65-1.75 (2m, 8 and 2H, respectively), 2.74 (t, *J* = 7.7 Hz, 2H), 6.36 (s, 1H), 7.68 (d, *J* = 8.9 Hz, 1H), 7.91 (dd, *J* = 8.9 Hz, ^4J = 2.3 Hz, 1H), 8.22 (d, ^4J = 2.3 Hz, 1H), 12.7 (s, br, 1H); ^{13}C-NMR (75 MHz, DMSO-d_6): δ 14.0, 22.1, 28.40, 28.46, 28.5, 31.2, 33.4, 107.3, 116.9, 121.1, 124.4, 126.5, 135.2, 138.8, 156.6, 173.4. HRMS calcd. (%) for $C_{16}H_{21}BrNO$: 322.0807; found: 322.0798.

Ethyl 2-heptyl-4-oxo-1,4-dihydroquinoline-6-carboxylate (**21**)
Orange solid; yield: 202 mg (13 %); m.p. = 197-198 °C; IR (KBr): ν 3261, 2926, 1719, 1645, 1495, 1278 cm^{-1}; ^{1}H-NMR (300MHz, DMSO-d_6) δ: 0.85 (3H, t, *J* = 6.7 Hz), 1.20-1.40 (11H, m), 1.60-1.75 (2H, m), 2.60 (2H, t, *J* = 7.6 Hz), 4.34 (2H, q, *J* = 7.2 Hz), 6.00 (1H, s), 7.61 (1H, d, *J* = 8.7 Hz), 8.12 (1H, dd, *J* = 8.6 Hz, ^4J = 2.0 Hz), 8.65 (1H, d, ^4J = 2.0 Hz), 11.74 (1H, bs); ^{13}C-NMR (150MHz, DMSO-d_6) δ: 14.0, 14.2, 22.1, 28.2, 28.4, 28.5, 31.2, 33.2, 60.8, 108.7, 118.5, 123.86, 123.92, 127.2, 131.4, 143.1, 154.6, 165.4, 176.8; HRMS calcd. (%) for $C_{19}H_{26}NO_3$: 316.1913; found: 316.1913.

6-Fluoro-2-heptylquinolin-4(1*H*)-one (**22**).
Pale yellow solid; yield: 357 mg (27 %); m. p. = 174-176 ºC (Et_2O); IR (KBr): □□3426, 1644, 1599 cm^{-1}; ^{1}H-NMR (300 MHz, DMSO-d_6): δ 0.85 (t, *J* = 6.7 Hz, 3H), 1.20-1.35, 1.60-1.75 (2m, 8 and 2H, respectively), 2.59 (t, *J* = 7.6 Hz, 2H), 5.95 (s, 1H), 7.53 (td, $^3J_{(H,H)}$ = $^3J_{(H,F)}$ = 8.7 Hz, $^4J_{(H,H)}$ = 2.9 Hz, 1H), 7.62 (dd, $^3J_{(H,H)}$ = 8.8 Hz, $^4J_{(H,F)}$ = 4.7 Hz, 1H), 7.69 (dd, $^3J_{(H,F)}$ = 9.4 Hz, $^4J_{(H,H)}$ = 2.9 Hz, 1H), 11.65 (s, br, 1H); ^{13}C-NMR (75 MHz, DMSO-d_6): δ 13.8, 22.0, 28.32, 28.33, 28.4, 31.1, 33.2, 106.9, 108.7 (d, $^2J_{(C,F)}$ = 22 Hz), 120.1 (d, $^2J_{(C,F)}$ = 25.4 Hz), 120.5 (d, $^3J_{(C,F)}$ = 8.4 Hz), 125.7 (d, $^3J_{(C,F)}$ = 6.4 Hz), 136.8 (d, $^4J_{(C,F)}$ = 0.6 Hz), 153.8, 158.1 (d, $^1J_{(C,F)}$ = 241 Hz), 176.0 (d, $^4J_{(C,F)}$ = 2.7 Hz). HRMS calcd. (%) for $C_{16}H_{21}FNO$: 262.1607; found: 262.1605.

6-(*tert*-Butyl)-2-heptylquinolin-4(1*H*)-one (**23**).
Pale yellow solid; yield: 385 mg (26 %); m. p. = 161-163 ºC (Et_2O); IR (KBr): □□3426, 1637, 1595, 1487 cm^{-1}; ^{1}H-NMR (300 MHz, DMSO-d_6): δ 0.86 (t, *J* = 6.7 Hz, 3H), 1.20-1.35 (m with s at 1.33, 17H), 1.60-1.75 (m, 2H), 2.57 (t, *J* = 7.6 Hz, 2H), 5.90 (s, 1H), 7.48 (d, *J* = 8.7 Hz, 1H), 7.71 (dd, *J* = 8.7 Hz, ^4J

= 2.3 Hz, 1H), 8.01 (d, ^{4}J = 2.3 Hz, 1H), 11.42 (s, br, 1H); ^{13}C-NMR (75 MHz, DMSO-d_6): δ 14.0, 22.1, 28.4 (2C), 28.5, 31.1 (3C), 31.2, 33.2, 34.4, 107.4, 117.7, 119.9, 124.1, 129.6, 138.2, 145.2, 153.2, 177.0. HRMS calcd. (%) for $C_{20}H_{30}NO$: 300.2327; found: 300.2318.

2-Heptyl-5,7-dimethylquinolin-4(1H)-one (**24**)

Pale yellow solid; yield: 612 mg (45 %); m.p. = 158-160 °C; IR (KBr): ν 3252, 2959, 1641, 1551, 1462, 1296 cm^{-1}; ^{1}H-NMR (300MHz, DMSO-d_6) δ: 0.85 (3H, t, *J* = 6.8 Hz), 1.15-1.40 (8H, m), 1.55-1.70 (2H, m), 2.31 (3H, s), 2.45-2.55 (2H, t, overlap with DMSO) 2.73 (3H, s), 5.75 (1H, s), 6.76 (1H, m), 7.09 (1H, s), 11.05 (1H, bs); ^{13}C-NMR (75MHz, DMSO-d_6) δ: 13.9, 21.0, 22.0, 22.9, 28.1, 28.35, 28.39, 31.1, 32.6, 109.2, 115.3, 120.9, 126.7, 138.8, 140.2, 142.0, 151.5, 179.4; HRMS calcd. (%) for $C_{18}H_{26}NO$: 272.2014; found: 272.2009.

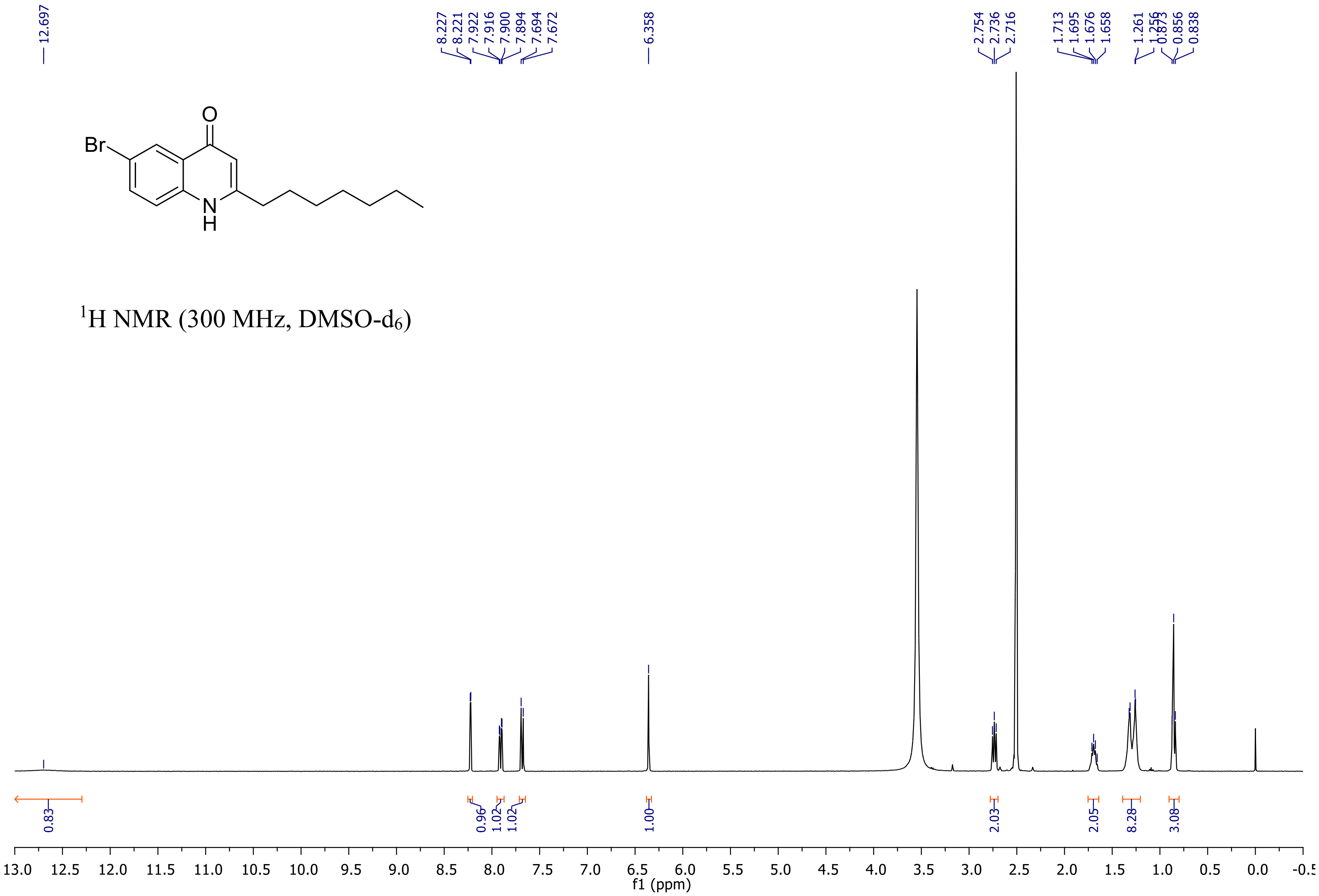

12.697
8.227
8.221
7.922
7.916
7.900
7.894
7.694
7.672
6.358
2.754
2.736
2.716
1.713
1.695
1.676
1.658
1.261
1.256
0.873
0.856
0.838
Br
O
N
H
^{1}H NMR (300 MHz, DMSO-d$_6$)
0.83
0.96
1.02
1.02
1.00
2.03
2.05
8.28
3.08
13.0 12.5 12.0 11.5 11.0 10.5 10.0 9.5 9.0 8.5 8.0 7.5 7.0 6.5 6.0 5.5 5.0 4.5 4.0 3.5 3.0 2.5 2.0 1.5 1.0 0.5 0.0 -0.5
f1 (ppm)

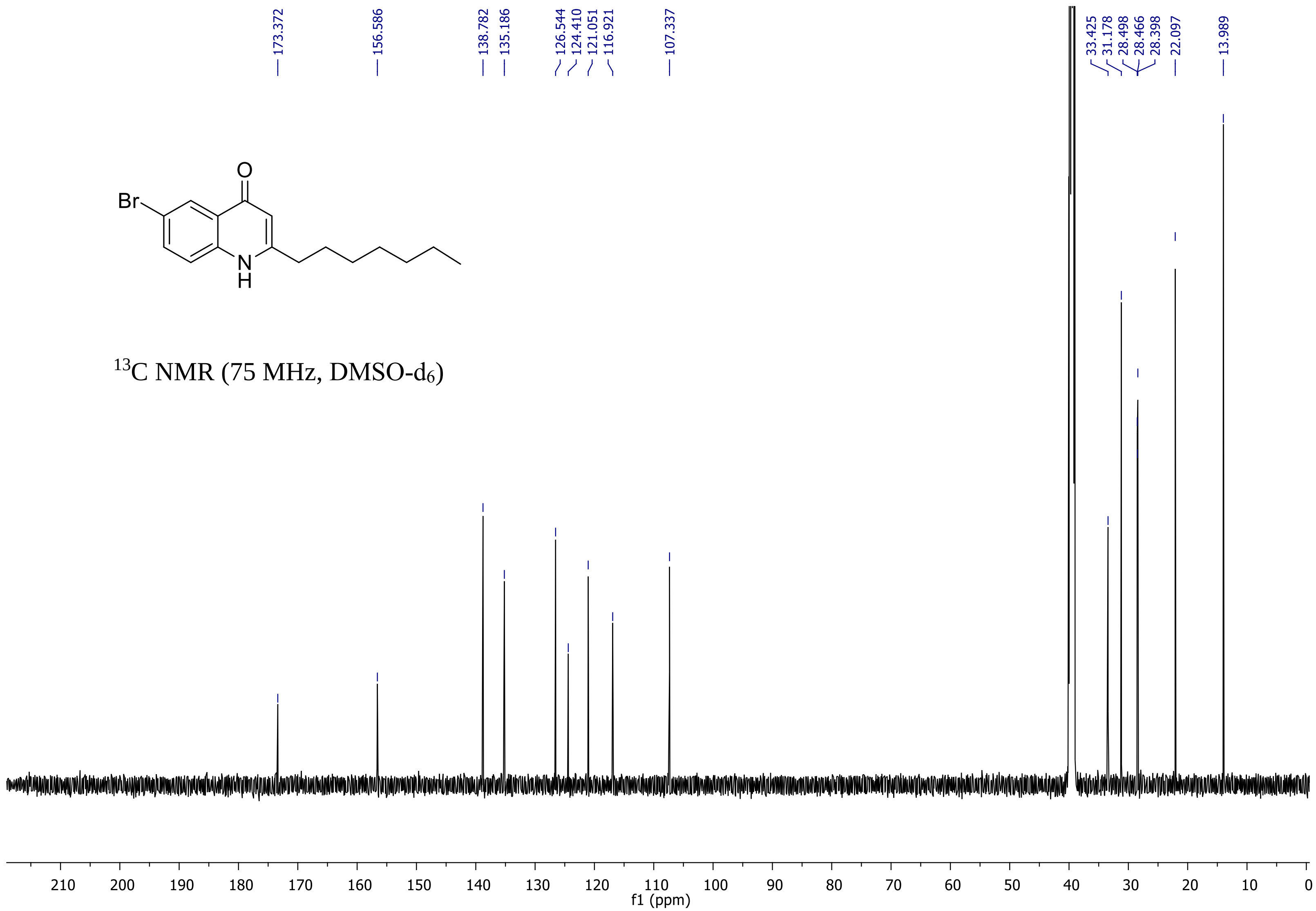
173.372
156.586
138.782
135.186
126.544
124.410
121.051
116.921
107.337
33.425
31.178
28.498
28.466
28.398
22.097
13.989
O
Br
N
H
13C NMR (75 MHz, DMSO-d6)
210
200
190
180
170
160
150
140
130
120
110
100
90
80
70
60
50
40
30
20
10
0
f1 (ppm)

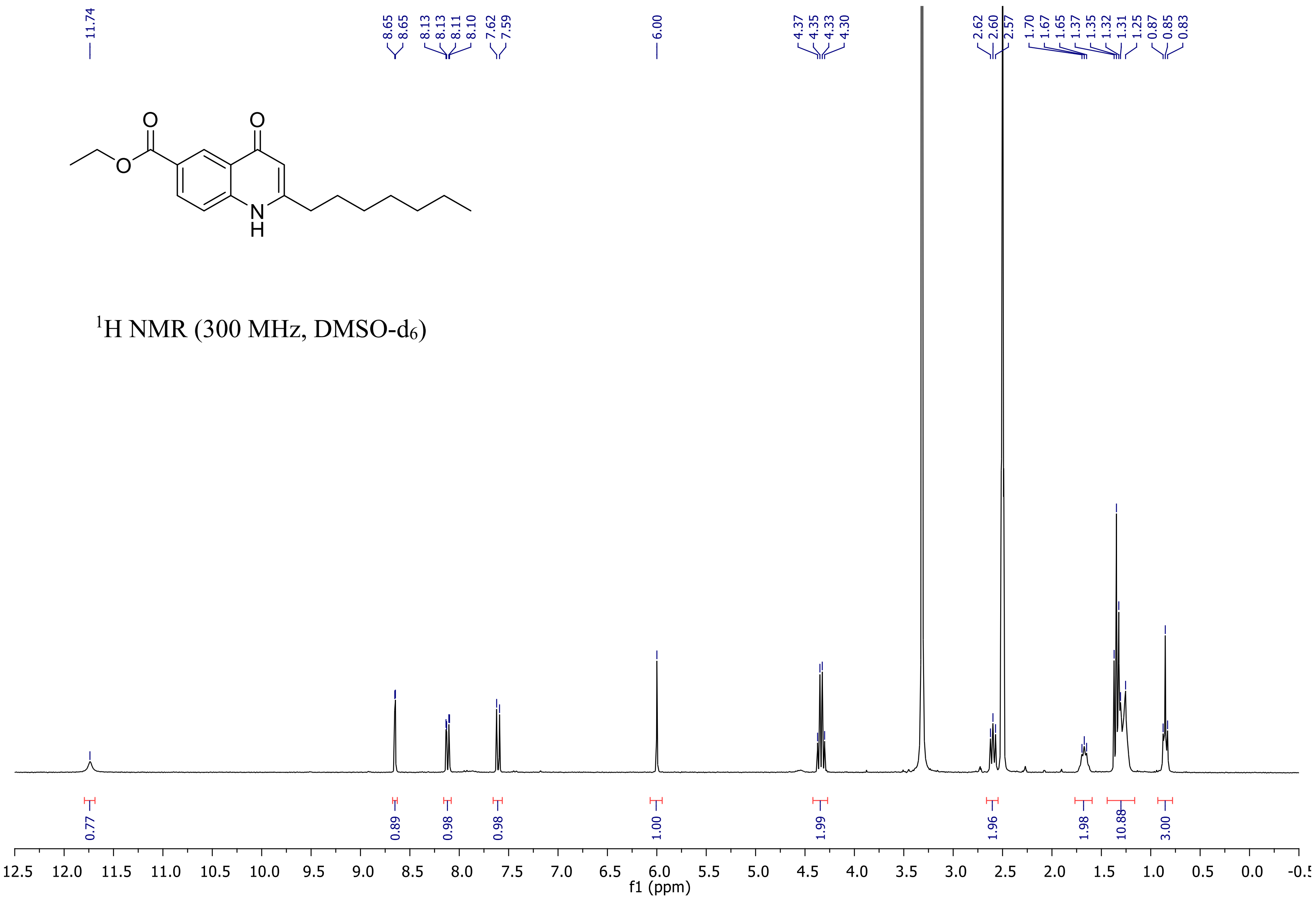

11.74
8.65
8.65
8.13
8.13
8.11
8.10
7.62
7.59
6.00
4.37
4.35
4.33
4.30
2.62
2.60
2.57
1.70
1.67
1.65
1.37
1.35
1.32
1.31
1.25
0.87
0.85
0.83
O
O
O
N
H
^{1}H NMR (300 MHz, DMSO-d$_6$)
0.77
0.89
0.98
0.98
1.00
1.99
1.96
1.98
10.88
3.00
12.5 12.0 11.5 11.0 10.5 10.0 9.5 9.0 8.5 8.0 7.5 7.0 6.5 6.0 5.5 5.0 4.5 4.0 3.5 3.0 2.5 2.0 1.5 1.0 0.5 0.0 -0.5
f1 (ppm)

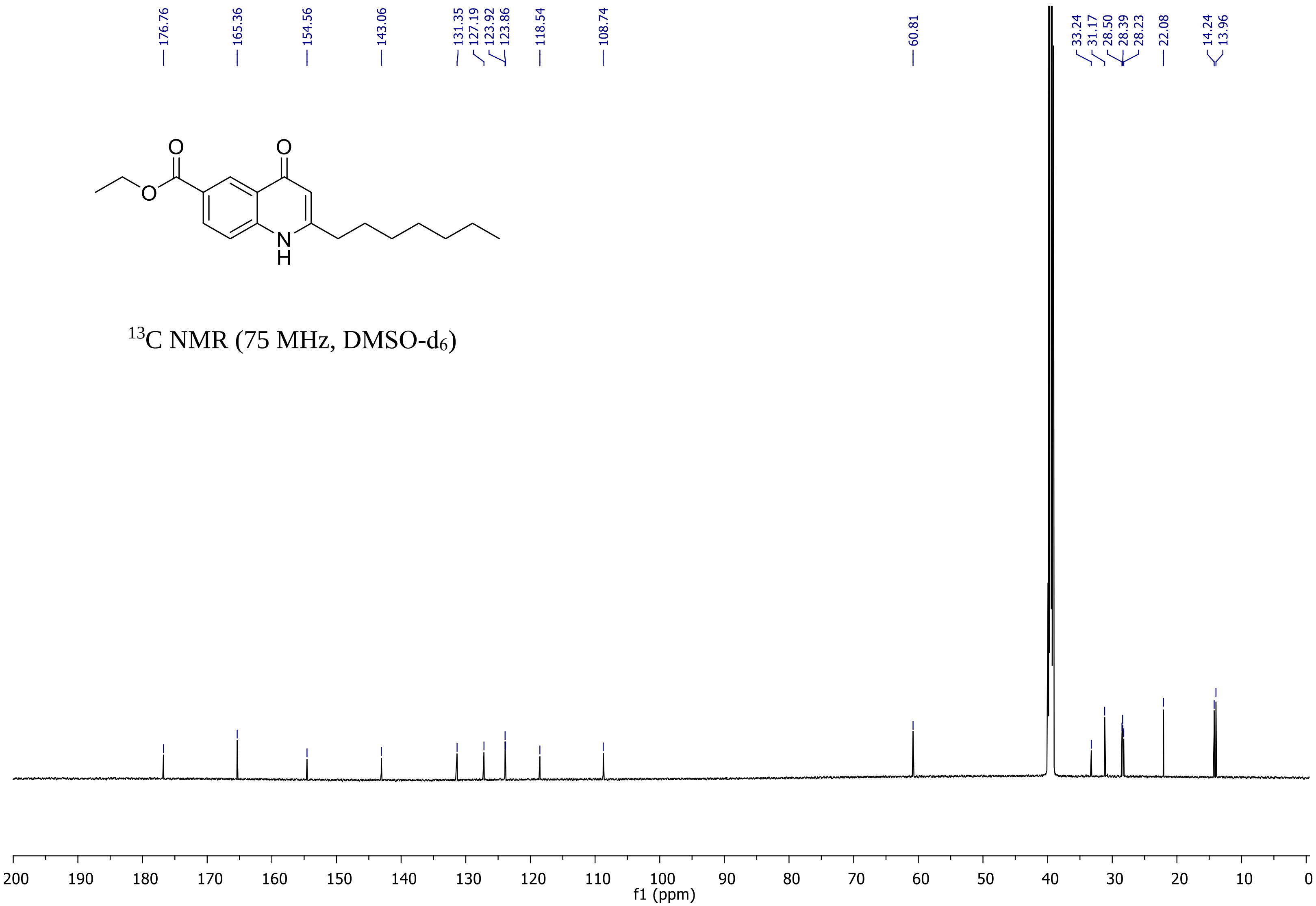
176.76
165.36
154.56
143.06
131.35
127.19
123.92
123.86
118.54
108.74
60.81
33.24
31.17
28.50
28.39
28.23
22.08
14.24
13.96
O
O
O
N
H
13C NMR (75 MHz, DMSO-d6)
200 190 180 170 160 150 140 130 120 110 100 90 80 70 60 50 40 30 20 10 0
f1 (ppm)

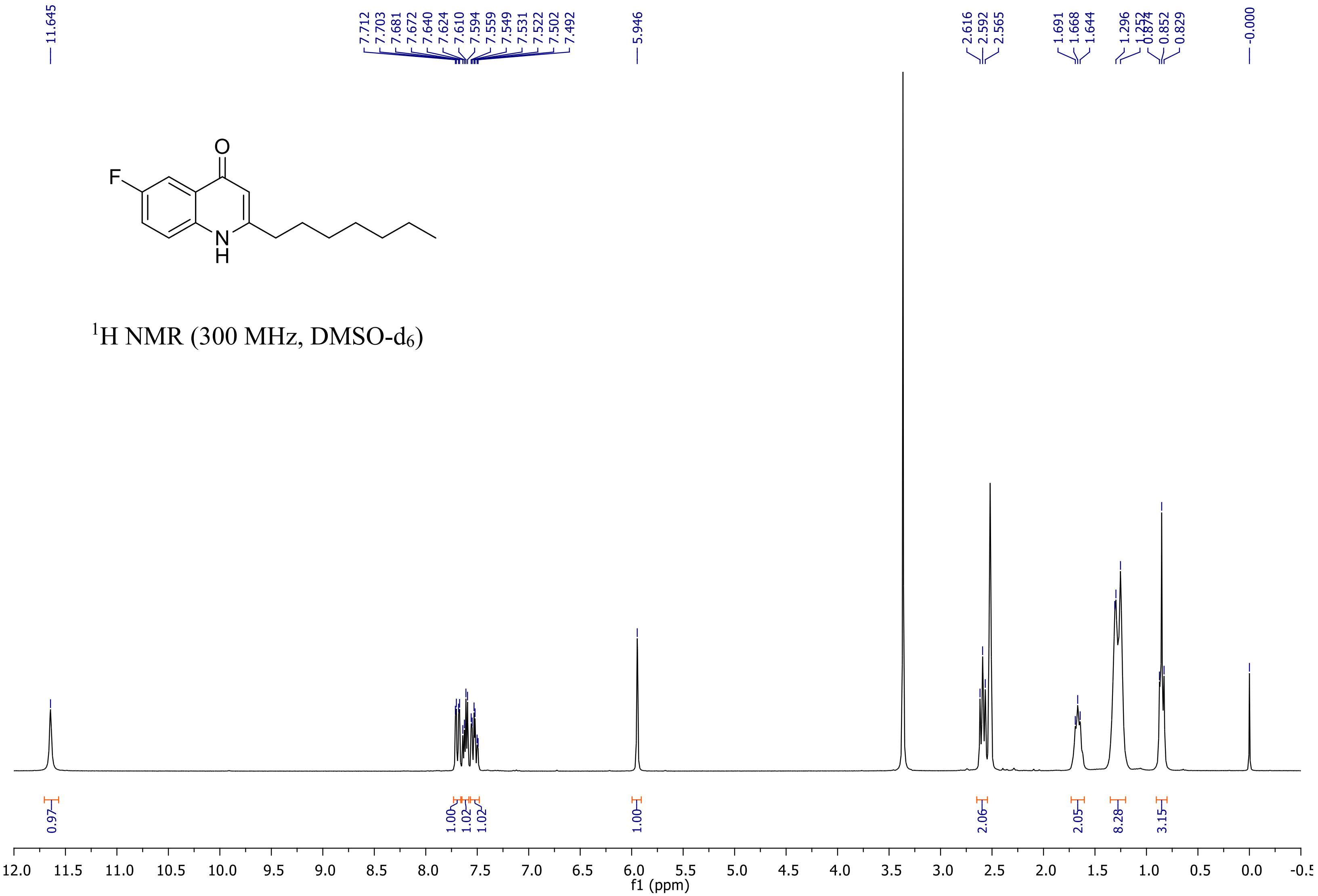

11.645
7.712
7.703
7.681
7.672
7.640
7.624
7.610
7.594
7.559
7.549
7.531
7.522
7.502
7.492
5.946
2.616
2.592
2.565
1.691
1.668
1.644
1.296
1.252
0.874
0.852
0.829
-0.000
O
F
N
H
^{1}H NMR (300 MHz, DMSO-d$_6$)
0.97
1.00
1.02
1.02
1.00
2.06
2.05
8.28
3.15
12.0
11.5
11.0
10.5
10.0
9.5
9.0
8.5
8.0
7.5
7.0
6.5
6.0
5.5
5.0
4.5
4.0
3.5
3.0
2.5
2.0
1.5
1.0
0.5
0.0
-0.5
f1 (ppm)

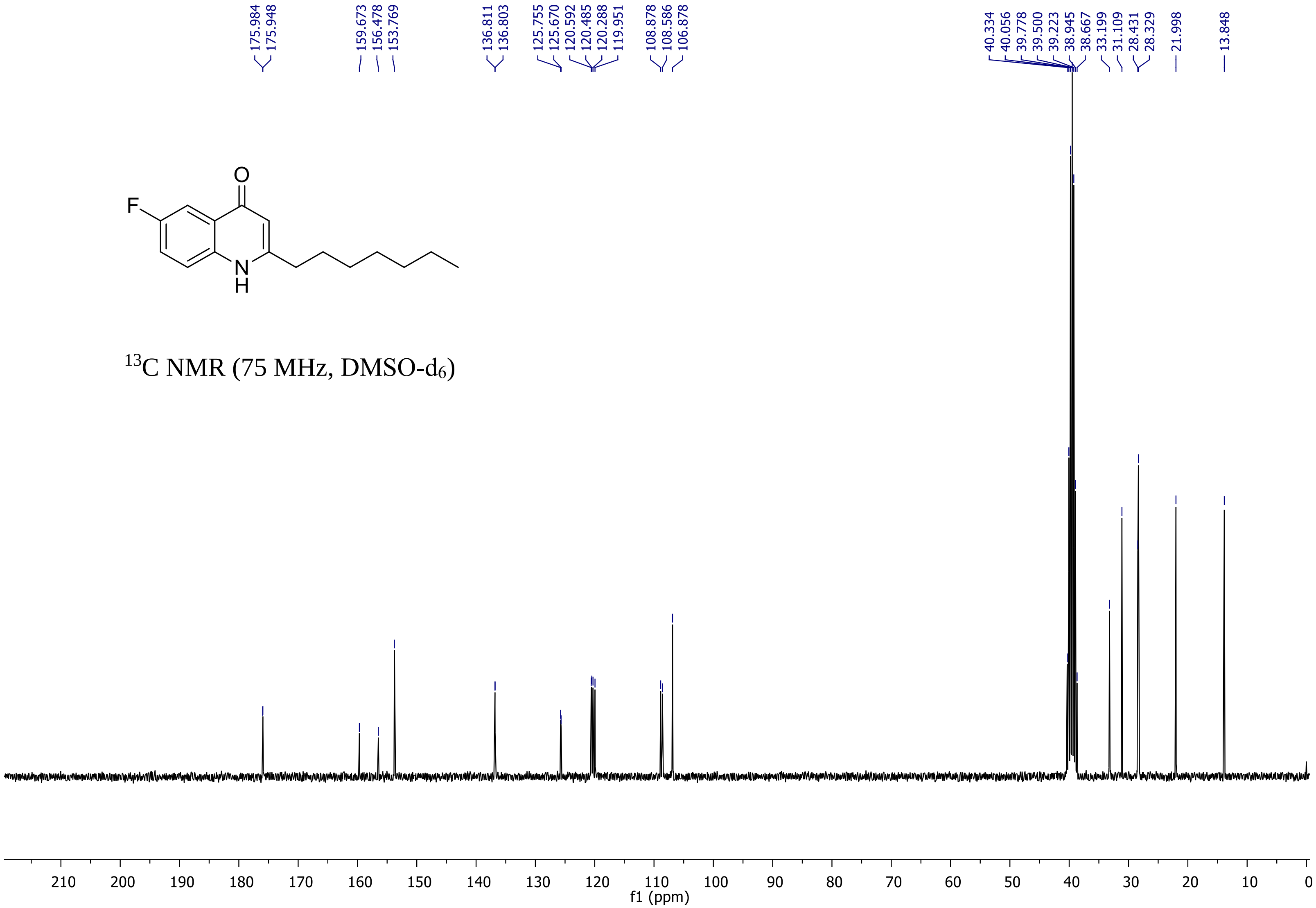

^{13}C NMR (75 MHz, DMSO-d_6)

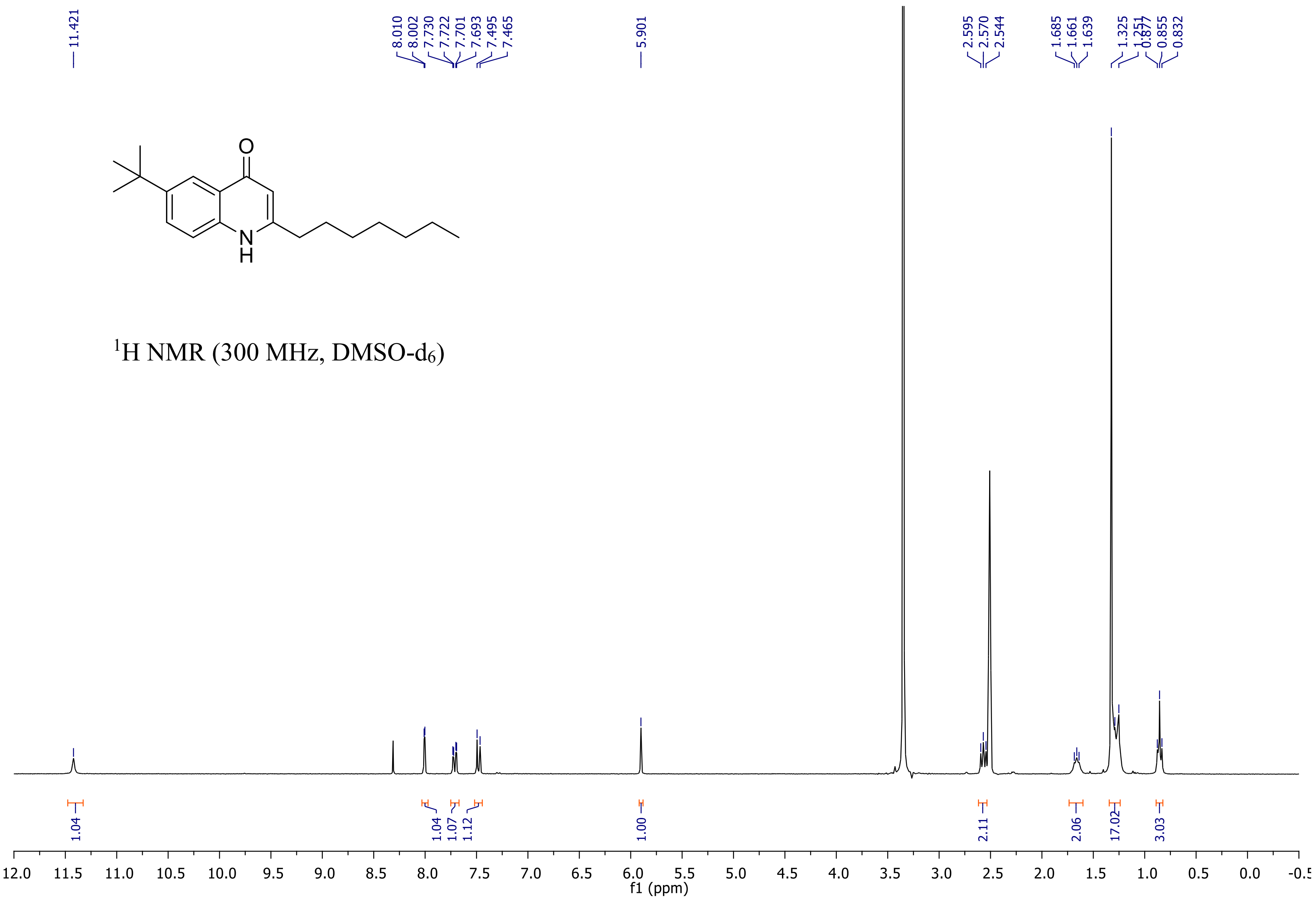

^{1}H NMR (300 MHz, DMSO-d_6)

^{13}C NMR (75 MHz, DMSO-d_6)

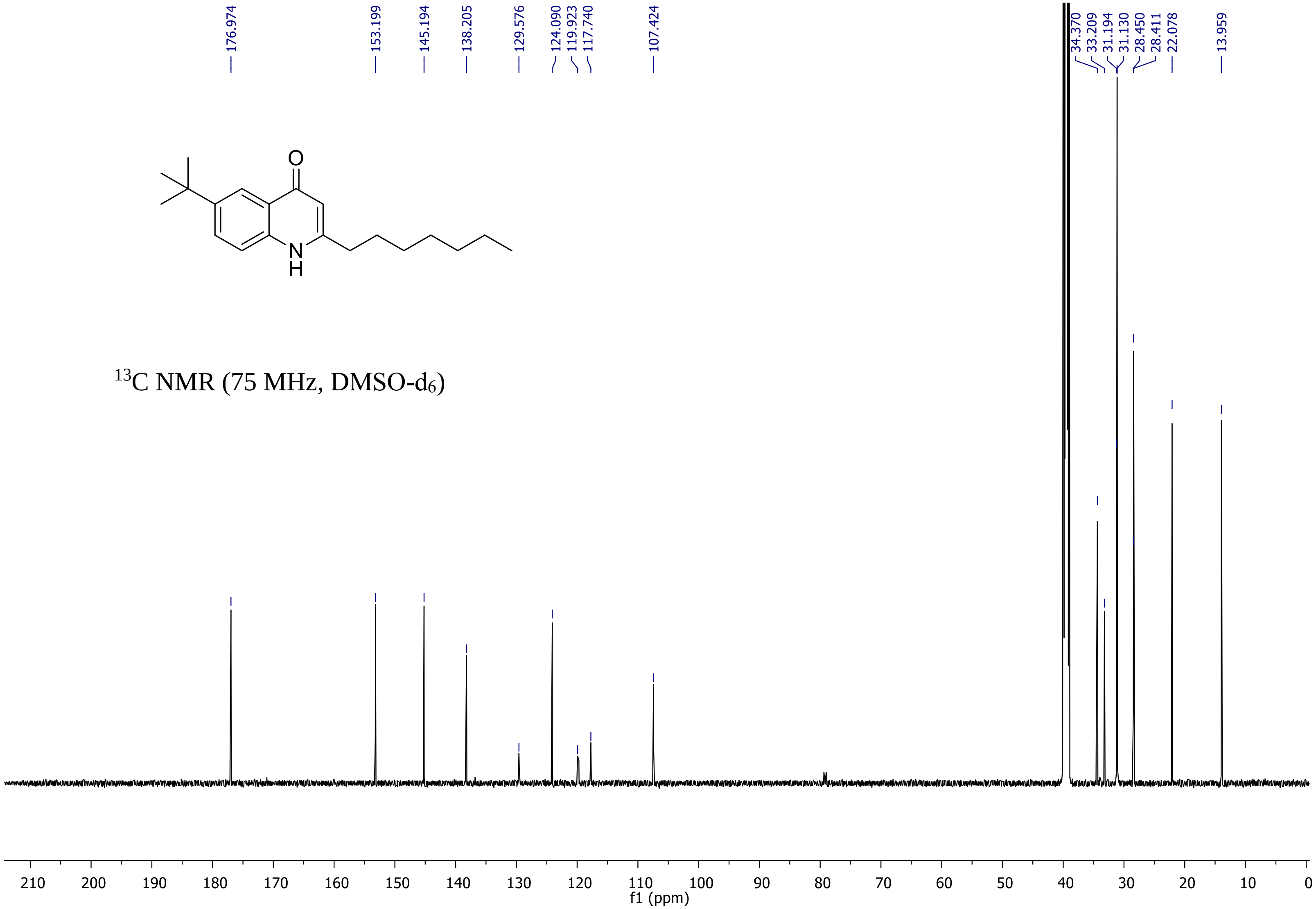

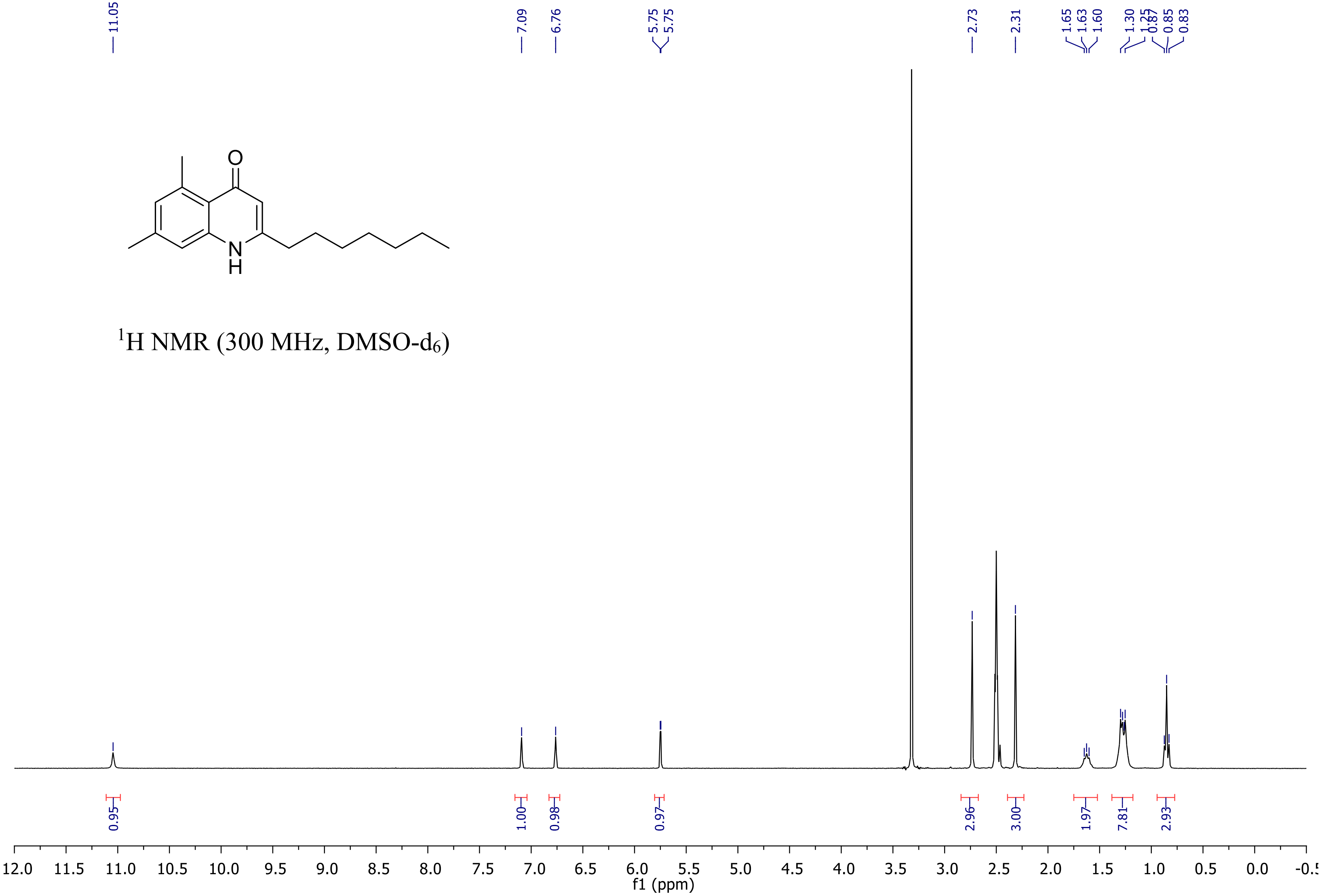

^{1}H NMR (300 MHz, DMSO-d_6)

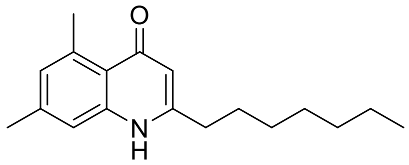

^{13}C NMR (75 MHz, DMSO-d$_6$)

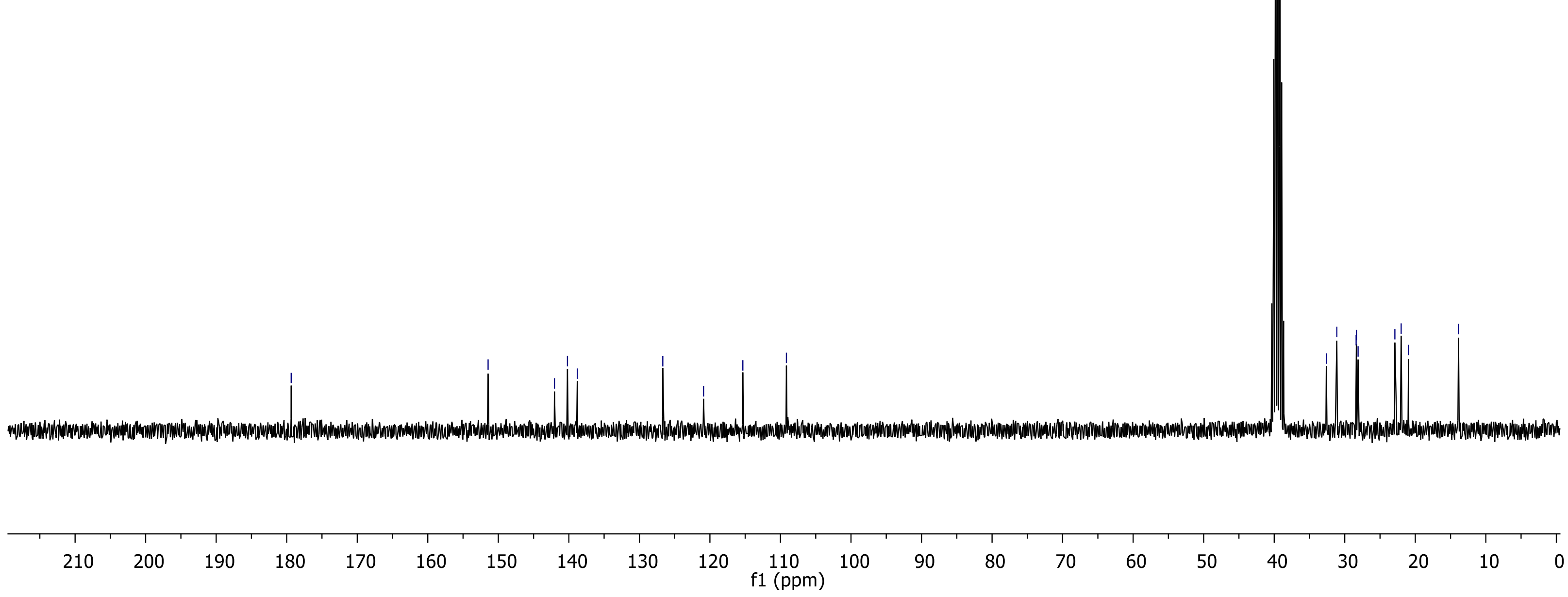

Supplement: Supplementary file 2 [file Data_Sheet_1.PDF]
