# Supplementary material for: Harnessing Bacterial Signals for Suppression of Biofilm Formation in the Nosocomial Fungal Pathogen Aspergillus fumigatus
Source: Front Microbiol. 2016 Dec 22;7:2074. doi: 10.3389/fmicb.2016.02074 (PMC5177741; doi:10.3389/fmicb.2016.02074)
Supplement: Supplementary file 4 [file Image_2.PDF]

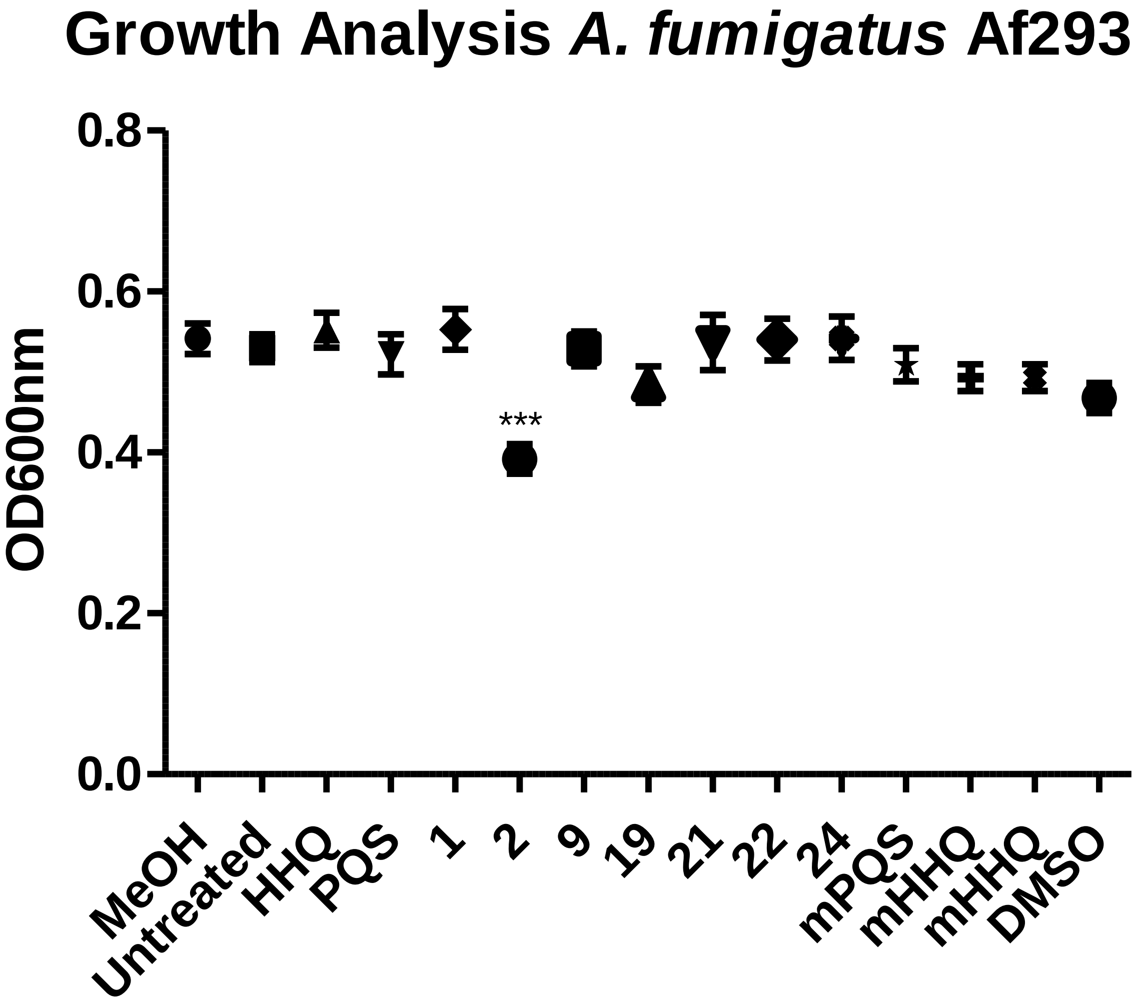

Figure S2. Growth analysis of *A. fumigatus* Af293 in the presence of lead compounds.
